# Antimicrobial susceptibility of *Brucella melitensis* in Kazakhstan

**DOI:** 10.1186/s13756-017-0293-x

**Published:** 2017-12-28

**Authors:** Alexandr Shevtsov, Marat Syzdykov, Andrey Kuznetsov, Alexandr Shustov, Elena Shevtsova, Kalysh Berdimuratova, Kasim Mukanov, Yerlan Ramankulov

**Affiliations:** 1 National Center for Biotechnology, Astana, Kazakhstan; 2 Kazakh Scientific Center of Quarantine and Zoonotic Diseases named by Masgut Aykimbayev, Almaty, Kazakhstan; 3 School of Science and Technology Nazarbayev University, Astana, Kazakhstan

**Keywords:** *Brucella melitensis*, Antimicrobial susceptibility, Kazakhstan

## Abstract

**Background:**

Kazakhstan belongs to countries with a high level of brucellosis among humans and farm animals. Although antibiotic therapy is the main way to treat acute brucellosis in humans there is still little information on a circulation of the antibiotic-resistant *Brucella* strains in the Central Eurasia. In this article we describe an occurrence of the drug resistance of *Brucella melitensis* isolates in Kazakhstan which is among the largest countries of the region.

**Methods:**

Susceptibilities to tetracyclin, gentamycin, doxycyclin, streptomycin and rifampicin were investigated in 329 clinical isolates of *Brucella melitensis* using E-test method.

**Results:**

All isolates were susceptible to streptomycin, tetracycline and doxycycline. 97.3% of the Brucella isolates were susceptible to gentamycin, although only 37.4% of isolates were susceptible to rifampicin. 21.9% of isolates had intermediate resistance, and 26.4% of isolates were resistant to this antibacterial drug.

**Conclusion:**

Isolates of *Brucella melitensis* circulating in Kazakhstan are susceptible to streptomycin, doxicyclin, tetracyclin and gentamycin. At the same time the resistance to rifampicin is widespread, almost half of the isolates were rifampicin-resistant (including the intermediate resistance).

## Background

Brucellosis remains the most common zoonotic disease with a worldwide incidence estimated at 500 thousand new cases per year [1]. The incidence may be underestimated because of diagnostic errors due to variability in clinical manifestations and underreporting and concealment of data [2, 3]. Infected terrestrial or marine mammals serve as natural reservoirs and infect people through direct contact or through consumption of the Brucella-contaminated livestock products [4]. Majority of brucellosis cases are recorded in the Mediterranean countries, Southern and Central America, Africa, Asia, Arabian Peninsula, Indian subcontinent, South-Eastern Europe and the Middle East region [1, 5]. Among the 12 recognized species in a genus *Brucella* [6], *B. melitensis*is is the most common and also the most virulent species which infect humans and causes a debilitating disease [7, 8].

Brucellosis is characterized by variability of clinical manifestations from absence of overt symptoms to multi-organ pathologies [9]. Antibiotics are commonly used to treat brucellosis and may suppress replication of the pathogen. One obstacle to the antibiotic therapy is that Brucella can survive in intracellular environments and replicate in macrophages and dendritic cells. For this reason the antibiotics must have intracellular activity. Also the most preferred antibiotics have low toxicity and are suitable for prolonged regiments [10]. The treatment regiments currently recommended by the WHO use a combination of doxycycline and rifampicin for 6 weeks, or doxycycline for 45 days and streptomycin for 21 days.

Brucella spp. actively circulates in humans and domestic animals. During the last 35 years in Kazakhstan an annual incidence rate of the disease in humans varied from 8.5 to 24 cases per 100 thousand (these rates averaged over the whole country) although in some endemic areas the incidence rates exceeded the figures significantly. The most prevalent species in the country in humans is *B. melitensis* [11]. Antibiotics play an important role in the strategy to treat the disease and predominantly determine effectiveness of the treatment. The widely used antibiotics for etiotropic treatment of brucellosis are tetracyclines, trimethoprim/sulfamethoxazole, aminoglycosides, rifampicin and fluoroquinolones [12, 13]. Low efficacy and frequent relapses after monotherapy led to a transition to a combined treatment regimen in 1986 [14]. However, the combination regimens also have a success below 100% because of a development of the resistance to the antibiotics [15].

In Kazakhstan the recommended therapeutic scheme is based on the WHO recommendations for the adult population, although the regiments for children and pregnant women may be changed if the drugs appear to be toxic. Despite a high incidence and defiant rate of relapses, still there is little information on the antibiotic resistance among Brucella strains circulating in Kazakhstan.

A high level of bacteriological hazard posed by the live *Brucella* requires complying to the BSL3 standards which makes an investigation of the antibiotic resistance cumbersome, expensive or even not possible. Nevertheless the data on the antibiotic resistance are important because the resistant *Brucella* may spread across wide regions [16–18]. In Kazakhstan and the neighboring regions studies of the antibiotic resistance of the circulating *Brucella* isolates can help to improve the efficacy of treatment.

In this paper we describe the results of a measurement of the antibiotic susceptibility in 329 *Brucella melitensis* isolates to five commonly used antibiotics.

## Methods

### Clinical isolates and characterization of *B. melitensis*

During 2008-2014 *Brucella* isolates were collected from patients which were seropositive to a *Brucella* antigen in a hemagglutination reaction with titers 1:200 and higher. Samples were collected from patients which presented in clinics with symptoms compatible with brucellosis. The blood for *Brucella* isolation was obtained during standard diagnostic procedures which require a bacteriological isolation of *Brucella* as a confirmatory test. The blood cultures were produced using the two-phase method proposed by Castaneda [19, 20]. Primary isolation and a species identification of the *Brucella* isolates were performed in local hospitals in 11 provinces of Kazakhstan. To confirm the identifications and for further investigation all isolates were further transferred to the Brucellosis laboratory in the Kazakh Scientific Center of Quarantine and Zoonotic Diseases (SC QZD). In total 329 isolates were collected in the SC QZD. The isolates were tested using standard procedures, e.g. CO_2_ requirement, production of H_2_S, reducing ability for aniline dyes (thionine and basic fuchsine at final concentrations 20-40 μg/ml), agglutination with specific antisera for A and M antigens and susceptibility to lysis with Tb and Weybridge phages. The species identification was confirmed using the “Bruce-ladder” multiplex PCR [21].

### Determination of minimum inhibitory concentrations (MICs)

MICs of tetracycline, gentamicin, doxycycline, streptomycin and rifampicin were determined using the E-test gradient strips (Biomerieux, Sweden). Suspensions of the bacteria with titers 10^5^-10^6^ CFU/ml were inoculated onto surfaces of Brucella agar plates with hemin and vitamin K (HiMedia Laboratories) and 5% sheep serum (Sigma-Aldrich). The MICs were determined following 48 h incubation in ambient air at 37 °C after placing of the E-test strips in the plates. The reference strains *E.coli* ATCC 25922 and *B. melitensis* 16 M were used to control performance of the test.

The MIC thresholds for tetracycline, gentamicin, doxycycline and streptomycin were calculated using the guidelines from the CLSI [22]. Since these guidelines do not provide a classification category for an “intermediate” resistance, all isolates which had the MIC value above the threshold were classified as resistant. Because the threshold for rifampicin has not been established against *Brucella* other guidelines suitable for slow-growing bacteria (*H.influenzae*) were used [23]. Isolates having the rifampicin MIC at a value 1.5 μg/mL were not classified as either susceptible or resistant because in the CLSI guidelines this value falls between the categories.

Different measures of the antibiotic effectiveness towards *Brucella* namely MIC50 and MIC90 were determined. The MIC50 and MIC90 are concentrations of the relevant antibiotics which inhibit growth of 50% of the isolates or 90% % of the isolates, respectively.

## Results

All 329 isolates were found to be a single species *B.melitensis* by their microbiological characteristics and by a multiplex PCR. Determined ranges of MICs, MIC50 and MIC90 and the assigned classification categories on the susceptibility/resistance are listed in Table 1.

**Table 1:** Ranges of MICs, MIC50 and MIC90 and antibiotic resistance in 329 *B. melitensis* isolates

	MICs range (μg/mL)	Susceptibility^a^ and thresholds (μg/mL)			MIC50 (μg/mL)		MIC90 (μg/mL)		Classification of isolates, No^1^ (%)^a^		
		S	I	R	Value	No^1^	Value	No^1^	S	I	R
rifampicin	0.38-16	≤1	2	≥4	1.5	170	8	326	123 (37.4)	72 (21.9)	87 (26.4)
rentamicin	0.5-8	≤4	–	–	1	188	3	318	320 (97.3)	–	9 (2.7)
streptomycin	0.25-4	≤8	–	–	0.75	191	3	327	329 (100)	–	–
tetracycline	0.032-0.5	≤1	–	–	0.094	165	0.25	324	329 (100)	–	–
doxycycline	0.023-0.19	≤1	–	–	0.047	212	0.094	319	329 (100)	–	–

^a^Definitions: S – susceptible, I- intermediate susceptibility, R – resistant; ^1^No – number of isolates

All 329 strains were susceptible to streptomycin, tetracycline and doxycycline and the mentioned antibiotics exhibited high antimicrobial activity. Also 97.3% of the isolates were susceptible to gentamicin, only 2.7% were resistant to gentamicin.

A high resistance rate was observed to rifampicin. Only 37.4% of the isolates were susceptible, 21.9% showed an intermediate susceptibility and 26.4% were resistant. Also 14.3% of the isolates which have MIC = 1.5 μg/mL were not classified into either category because the CLSI guidelines do not allow to unambiguously assign the category. One striking observation is that of the nine isolates resistant to gentamycin eight are also resistant to rifampicin.

The MIC90 values for tetracycline and doxycycline were below the respective CLSI thresholds (25% and 9.4% of the thresholds, respectively). For streptomycin the MIC90 was 37.5% of the threshold and for gentamicin the MIC90 was of 75% of the threshold. At the same time, for rifampicin even the MIC50 exceeded the threshold indicating low susceptibility to rifampicin in the circulating *Brucella* isolates.

Occurrence of resistant isolates differed in samples from various regions of Kazakhstan. All nine isolates which were resistant to gentamicin were collected from the South Kazakhstan. In contrast, the isolates resistant to rifampicin were found almost throughout the country: samples from 7 out of 11 provinces of Kazakhstan contained the rifampicin-resistant isolates. In the particular provinces the occurrence of the rifampicin-resistant isolates is particularly high: 73.3% in Kyzylorda, 57.1% in Zhambyl, 52.9% in Atyrau and 50% in Karaganda provinces. The same provinces also have the highest prevalence of stains with the intermediate of high resistance to rifampicin (Table 2).

**Table 2:** Geographical distribution of *Brucella* isolates and their susceptibility/resistance to gentamicin and rifampicin

Region	Total number of isolates (T)	Susceptibility to gentamicin				Susceptibility to rifampicin					
		S (T)	S (%)^a^	R (T)	R (%)^a^	S (T)	S (%)	I (T)	I (%)^a^	R (T)	R (%)
Akmola	1	1	100	0.0	0.0	1	100.0	0.0	0.0	0.0	0.0
Aktibinsk	7	7	100	0.0	0.0	1	14.3	3	42.9	0.0	0.0
Almaty	91	91	100	0.0	0.0	39	42.9	22	24.2	11	12.1
Atyrau	17	17	100	0.0	0.0	6	35.3	2	11.8	9	52.9
East-Kaz. region	26	26	100	0.0	0.0	7	26.9	11	42.3	5	19.2
Zhambyl	49	41	83.7	8	16.3	4	8.2	11	22.4	28	57.1
West-Kaz. region	26	26	100.0	0.0	0.0	24	92.3	2	7.7	0.0	0.0
Karagandy	16	16	100.0	0.0	0.0	1	6.3	5	31.3	8	50.0
Kyzylorda	30	30	100.0	0.0	0.0	3	10.0	4	13.3	22	73.3
North-Kaz. region	2	2	100.0	0.0	0.0	0.0	0.0	1	50.0	0.0	0.0
South-Kaz. region	64	63	98.4	1	1.6	37	57.8	11	17.2	4	6.3

^a^S(%) = S/T × 100; R (%) = R/T × 100; I (%) = I/T × 100

## Discussion

In our study we show that *Brucella melitensis* isolates in Kazakhstan are susceptible to streptomycin, doxicyclin and tetracyclin, and an absolute majority of the isolates are also susceptible to gentamycin. A very different susceptibility rate was found to rifampicin. The intermediate and full resistance to rifampicin was found in 21.9% and 26.4%, respectively. This is an important observation because rifampicin is used as the frontline antibiotic in 9 out of 17 therapeutic schemes for brucellosis in Kazakhstan. Also unexpectedly out of nine isolates resistant to gentamicin eight isolates are also resistant to rifampicin.

In a search for a reason for the high occurrence of rifampicin resistance among *Brucella* isolates in Kazakhstan we compared our data with known geographical patterns of rifampicin resistance for other bacterial pathogens. We speculate that the resistance in *Brucella* develops in conjunction to a rise in multidrug-resistant tuberculosis (MDR-TB). Kazakhstan is among the top eight countries in the world by an incidence of MDR-TB. Also a frequency of MDR-TB is annually growing among the newly diagnosed TB cases [24]. The highest incidence of MDR-TB per 100,000 population is registered in the Atyrau, Kyzylorda, East Kazakhstan, West Kazakhstan, Almaty and Zhambyl provinces [25]. The same provinces have the highest occurrence of the resistance to rifampicin.

It was discussed earlier that in regions with prevalent brucellosis there is a potential of developing of MDR-TB because of a treatment of both diseases with the same frontline antibiotics [26]. In reality, the provinces of Kazakhstan with high rates of MDR-TB also have high rates of *Brucella* resistance to rifampricin [27–29]. Among other countries of the world only Kyrgyzstan (which borders Kazakhstan) also has the high rates of both MDR-TB and brucellosis. Supposedly, the rifampricin treatment of brucellosis is not the only reason for an increase of MDR-TB in the mentioned countries. In Kazakhstan the treatment of brucellosis is carried out only in inpatient clinics thus ensuring compliancy to a treatment regimen. Among possible reasons for the appearance of the antibiotic-resistant *Brucella* isolates is an uncontrolled use of antibiotics without prescription in a population to treat influenza-like symptoms and also uncontrolled distribution of the antibiotics by local sellers (this practice was stopped by an issuance of novel regulations on sells of the antibiotics in 2016). Data on a previous treatment of TB in the population from which *Brucella* isolates were obtained are not available.

Our study underscores a need in a comprehensive and regular monitoring of the antibiotic resistance in the circulating *Brucella* isolates. Given that brucellosis is a zoonotic disease there is a need to expand the studies to include the isolates from animals. The data presented in this article will help in understanding and ensuring of an adequate control on the brucellosis in the Central Eurasia.

## Conclusion

This study shows that 48% of isolates of *Brucella mellitensis* collected from humans in Kazakhstan are resistant to rifampicin. The data reported can be used to optimize the therapy regiments.

## Abbreviations

ATCC: American type culture collection
CFU: Colony forming unit
CLSI: Clinical & Laboratory Standards Institute
MIC: Minimum inhibitory concentration
PCR: Polymerase chain reaction
SC QZD: Kazakh Scientific Center of the Quarantine and Zoonotic Diseases
